# Cancer-Related Fatigue in Post-Treatment Cancer Survivors: Theory-Based Development of a Web-Based Intervention

**DOI:** 10.2196/cancer.6987

**Published:** 2017-07-04

**Authors:** Teresa Corbett, Jane C Walsh, AnnMarie Groarke, Rona Moss-Morris, Eimear Morrissey, Brian E McGuire

**Affiliations:** ^1^ Centre for Clinical and Community Applications of Health Psychology (CCCAHP) School of Psychology Faculty of Social, Human and Mathematical Sciences Southampton United Kingdom; ^2^ School of Psychology NUI Galway Galway Ireland; ^3^ Health Psychology Section Psychology Department, Institute of Psychiatry King's College London London United Kingdom

**Keywords:** cancer, survivor, design, person-based approach, theory

## Abstract

**Background:**

Cancer-related fatigue (CrF) is the most common and disruptive symptom experienced by cancer survivors. We aimed to develop a theory-based, interactive Web-based intervention designed to facilitate self-management and enhance coping with CrF following cancer treatment.

**Objective:**

The aim of our study was to outline the rationale, decision-making processes, methods, and findings which led to the development of a Web-based intervention to be tested in a feasibility trial. This paper outlines the process and method of development of the intervention.

**Methods:**

An extensive review of the literature and qualitative research was conducted to establish a therapeutic approach for this intervention, based on theory. The psychological principles used in the development process are outlined, and we also clarify hypothesized causal mechanisms. We describe decision-making processes involved in the development of the content of the intervention, input from the target patient group and stakeholders, the design of the website features, and the initial user testing of the website.

**Results:**

The cocreation of the intervention with the experts and service users allowed the design team to ensure that an acceptable intervention was developed. This evidence-based Web-based program is the ﬁrst intervention of its kind based on self-regulation model theory, with the primary aim of targeting the representations of fatigue and enhancing self-management of CrF, speciﬁcally.

**Conclusions:**

This research sought to integrate psychological theory, existing evidence of effective interventions, empirically derived principles of Web design, and the views of potential users into the systematic planning and design of the intervention of an easy-to-use website for cancer survivors.

## Introduction

The number of posttreatment cancer survivors in Ireland is set to increase in coming years due to advances in screening and treatment [1,2]. This group will require ongoing supportive care as many will experience persistent negative side-effects that can impair the quality of life. Cancer-related fatigue (CrF) is the most common and disruptive symptom experienced by cancer survivors. Fatigue is extremely complex and likely to involve the interaction of several physiologic and psychological mechanisms. Current evidence supports the use of nonpharmacological treatment strategies for reducing CrF [3]. Web-based interventions have been shown to be an effective mode of delivery and can facilitate self-management of long-term conditions [4,5], including CrF [6-9]. Chou, Liu, Post, and Hesse [10] encourage using the Internet to better serve survivors’ needs as it is increasingly being used as a resource by cancer survivors. Internet delivery overcomes isolation of time, mobility, and geography [11] that are sometimes cited as barriers to seeking support for CrF [12]. Web-based interventions allow participants to engage with the content an infinite number of times, at their own pace, and in the comfort of their chosen environment [13]. Such interventions may, therefore, increase access for users by providing 24-hour access to health care interventions and having the potential to reach huge numbers of people [11]. Use of such tools may enhance empowerment and effective self-management of fatigue [6,8].

This paper describes the development of a theory-based, interactive Web-based intervention designed to facilitate self-management and enhance coping with CrF following cancer treatment [14]. There has been an increase in the development of eHealth interventions; however, these are often are not clearly described in sufficient detail to allow for replication [15,16]. Furthermore, many of these interventions are frequently not based explicitly on a particular theory or therapy [17,18]. This paper outlines the process and method of development to allow readers to gain an insight into the intervention itself but also to provide a template for developing other interventions. The content and principles used in the development process are described [19], while also clarifying hypothesized causal mechanisms [20]. The description of the design process is presented in 4 sections. The first section describes the process of establishing a therapeutic approach based on theory. The second section describes the design of the content of the intervention. The third part describes the design of the website features. The final section describes the initial usability testing of the website. The aim is to outline the rationale, decision-making processes, methods, and findings which led to the development of a Web-based intervention to be tested in a feasibility trial [21].

## Methods

In this section we outline the research and planning approaches we used to develop the content of the intervention.

### Part 1: Establishing a Therapeutic Approach Based on Theory and Evidence

The underlying aetiology of CrF is not well understood [22] but it is thought to be a multidimensional symptom associated with physical, mental, and emotional factors. The processes that cause persistent fatigue remain unclear [23].

Biological factors such as cancer and its treatment may lead to initial fatigue during cancer [24]. Fatigue during treatment is associated with an inflammatory response to cancer and its treatment. However, during survivorship, it is proposed that cognitive-behavioral factors may maintain fatigue [25]. These include cognitive or emotional responses to the fatigue and coping strategies employed.

Interventions for fatigue based on cognitive behavioral therapy (CBT) aim to address cognitions, emotions, behaviors, or a combination of these [26]. CBT has been found to be effective for fatigue associated with other conditions [27-29] and may be more effective than alternative psychological therapies in reducing fatigue symptoms [30].

Theoretically, the therapeutic techniques used in CBT are comparable with constructs outlined in the self-regulation model proposed by Leventhal [31,32]. Using qualitative research, we concluded that the self-regulation model to describe fatigue after cancer provides an integrated theoretical model for developing interventions for fatigue-based on cognitive-behavioral principles [33]. This theory could clarify the processes by which CBT can impact posttreatment CrF by outlining the mechanisms that are hypothesized to bring about change in symptoms [34-36].

Interventions which target these processes may improve symptom management in CrF [37]. In our intervention, the aim was to help the participant engage in a process of appraising their representation of the fatigue symptoms, and also help them to identify adaptive coping strategies hypothesized to mediate change in fatigue outcomes [14,33].

#### Drawing on Existing Evidence

##### Systematic Review

In order to identify therapies that are likely to be most effective for fatigue after cancer, a systematic review of psychological interventions was conducted. The systematic review and meta-analysis found an overall positive effect of psychological interventions on fatigue in cancer survivors [38]. However, there was considerable heterogeneity, not only in design and outcomes, but also in the quality and usability of the specific interventions. The review identified 5 primary psychological intervention types including CBT, psychoeducation, mindfulness-based strategies, motivational interviewing, and supportive therapies. Since no single intervention type emerged as superior in this review, a decision was made to base the current intervention on CBT. This decision was based on the quality and quantity of existing literature and theory [39].

##### Similar Interventions

Similar interventions were consulted to facilitate selection of specific behaviors that would be targeted in the intervention [34,40]. The structure and layout was compiled in line with previous CBT interventions, in particular, the Web-based “MS Invigor8” intervention [41] and the “Understanding and managing persistent cancer-related fatigue” manual [42]. MS Invigor8 was developed from a therapist-delivered, CBT-based manualized self-management intervention shown to be an effective treatment for multiple sclerosis (MS) fatigue in a randomized controlled trial [43]. The original manual was based on a cognitive behavior model of fatigue in MS [44]. A pilot trial of the Web-based version (MS Invigor8: Breaking the cycle of fatigue) suggests that a Web-based version with minimal telephone support may be a cost-effective way of delivering the intervention for MS fatigue [41]. “Understanding and managing persistent cancer-related fatigue” is a manual structured on CBT techniques and addresses issues such as inactivity, low mood, sleep problems, worry, and reclaiming life after cancer [42]. This manual was developed for Irish individuals with fatigue after cancer but has not been tested for effectiveness. Further information and specific components of the intervention were also informed by the available evidence on symptom-focusing [45]; activity scheduling, insomnia management [46-48]; and stress management [49] in cancer patients. Relaxation techniques and descriptions on activity pacing from the “Feeling better” manual were also incorporated [50].

##### Practice Guidelines

Existing practice recommendations were also consulted to assess the applicability of CBT for this participant group. The National Comprehensive Cancer Network has published guidance on supporting patients with CrF following treatment. Recommendations include the use of CBT [51]. CBT is also recommended by the American Cancer Society or American Society of Clinical Oncology Breast Cancer Survivorship Care guidelines [52].

### Part 2: Designing the Content of the Intervention

**Figure 1:**
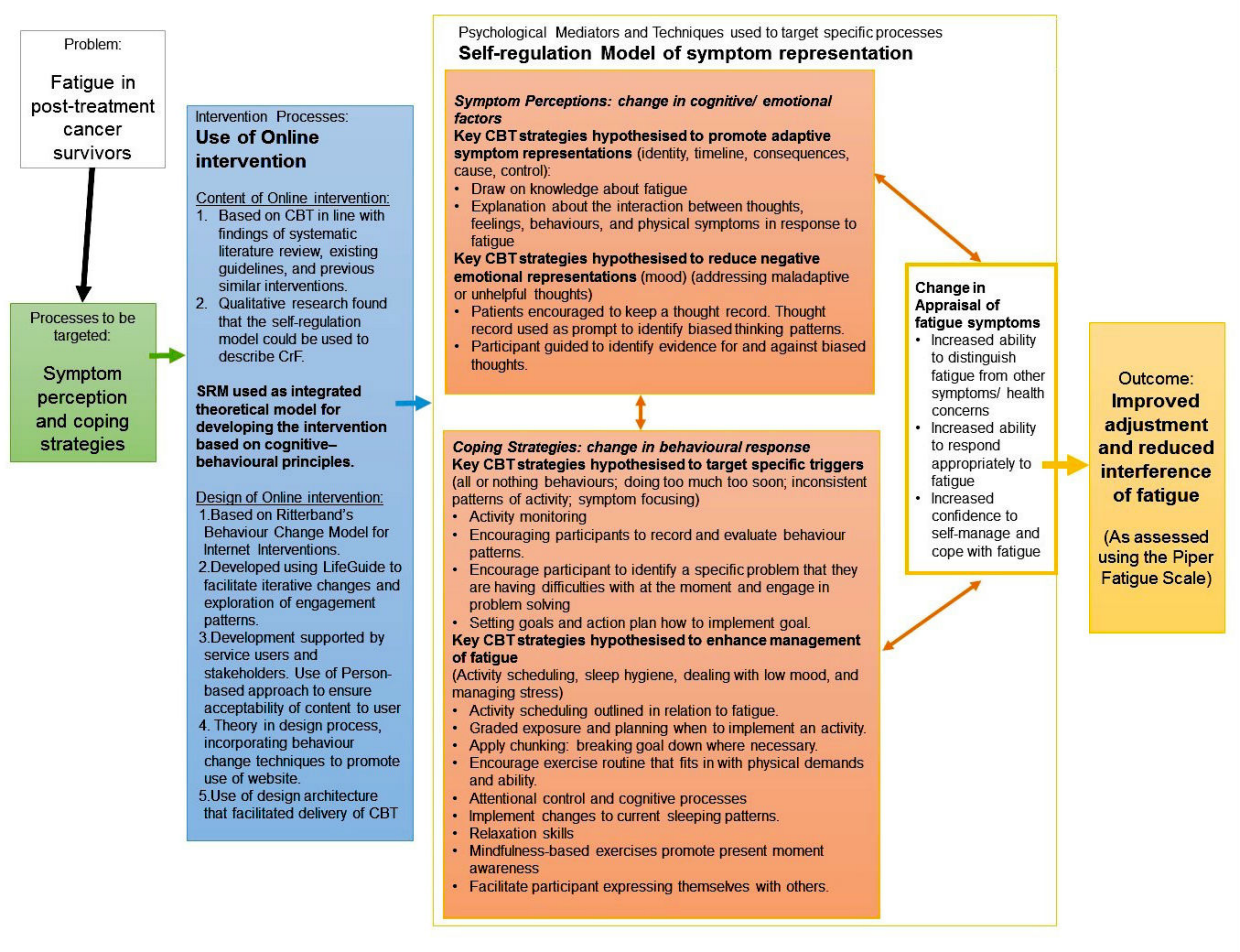
Logic model which includes theoretical model, the processes to be targeted, interventions to be used to target specific processes, and outcomes to be used in an efficacy randomized controlled trial (RCT).

An intervention content manual was developed in line with previous literature and existing guidelines. The content of this intervention draws upon established cognitive-behavioral models of fatigue as well as the self-regulation model of health and illness [33]. A logic model based on the findings of the systematic review, qualitative interviews, and the feasibility study is illustrated in Figure 1. Hypothesized influences on behavior were linked to intervention sessions that were established based on previous research and CBT guidelines [22,27,41,45,53]. It is hypothesized that certain key CBT techniques are likely to influence symptom representation and coping with fatigue and that an intervention addressing these factors is likely to change an individual’s appraisal of symptoms and coping responses. Changes in symptom appraisal and coping are hypothesized to lead to improvements in adjustment to, and interference of, fatigue [14].

Once the content manual was developed based on traditional CBT programs, the behavior change technique (BCT) taxonomy (v1) was employed to describe components of the intervention [30]. To ensure that a comprehensive description of all aspects of the intervention was provided, content was also described with reference to the CBT competence framework for working with people with persistent physical health conditions [53]. We then summarized each of the intervention sessions and their association with the CBT [53], and the self-regulation model [54,55] constructs targeted and the BCTs used [14].

The use of the BCT taxonomy (v1) was not intended to reflect the effectiveness of particular BCTs in this intervention [35], but rather as a tool to specify techniques of the CBT intervention as a whole. The content of each of the sessions was analyzed independently by 2 coders (TC and EM). TC developed the content. EM was naïve to the content, theoretical basis, or aims of the intervention. BCTs were coded with a “0” if absent and a “1” if present. The interrater reliability was found to be moderate across each of the sessions (average κ=.67, *P*<.01; See Table 1). Sixty different BCTs were present across the sessions. The sessions increased in complexity, with the number of BCTs increasing as the intervention progressed. The session with most BCTs was session 5. The most commonly used BCT within the sessions was “13.2. Framing or reframing” which featured in every session.

**Table 1:** Interrater reliability of behavior change technique (BCT) coding for each session.

Session	Kappa
Session 1	.592
Session 2	.692
Session 3	.671
Session 4	.608
Session 5	.754
Session 6	.688
Session 7	.669
Session 8	.668
Average	.668

#### Preferences of Potential Users Sought in Design: Incorporating a Person-Based Approach

Once theoretical foundations and preliminary content was mapped out, qualitative research was used to gain insight into user characteristics and identify the preferences of potential users [13,56]. The aim was to adapt the developed content for delivery via the Internet. Focus groups were carried out with survivors of cancer with fatigue (N=18), to explore their representations of fatigue in order to test the application of the theoretical model as proposed [33]. In a separate focus group session, the same participants were also asked about their perceptions of Web-based interventions and the type of features that were viewed as acceptable or unacceptable.

Participants highlighted a need for support throughout a Web-based intervention, particularly during this transitionary stage after treatment [57]. eHealth interventions can be enhanced by the use of additional methods of communicating with participants. Social support was offered through the intervention provider rather than providing a social networking facility for participants. This was considered to be better suited to this intervention where resources were limited given the inconsistency regarding the credibility and benefits of social support interventions and difficulties associated with ensuring that these tools were appropriately engaged with [58]. Messages of encouragement were used to stimulate adherence. It was decided that the research team would call participants half-way through the trial. A semistructured interview guide was developed and outlined in a manual to enable replication. These phone calls would support participants with any problems with the sessions or content, while also allowing participants to discuss their thoughts about CBT, their progress, and any of the messages provided in the intervention. The calls would be audio recorded and checked for fidelity, and any relevant content would be used to guide improvements for future iterations of the website [14]. Contact was otherwise provided via regular email reminders and updates about the intervention.

Developing a Web-based resource to support self-management after cancer treatment was endorsed by the majority of participants. Important contributions were made by participants regarding the need for some degree of personalization, credibility, and recognition of the fatigued nature of those using the website. Drawing on personal experiences, participants highlighted important domains such as an emphasis on moving forward with life after cancer rather than focusing too much on the illness.

Participants requested that the website focus on what they are able to do rather than on the limitations imposed on them by their fatigue. Therefore we aimed to increase individuals’ perceptions of their capability to change behavior rather than pointing to the implications of not changing [59]. Participants are congratulated on milestones throughout the program and emails include verbal persuasion to continue with the program [14].

Individuals emphasized the need to develop an attractive and engaging program. The name “Refresh” was chosen as suggested by participants in the qualitative research. This word was to reflect a new beginning (ie, a fresh start), and a focus on what people could do rather than the cancer experience. In order to ensure that the website was attractive to users, we sought to ensure that a simple, clear design was used [60]. Therefore, aspects such as appearance and the use of color were considered throughout the design process. The color-scheme throughout reflected the affiliation with the University.

Individuals emphasized the need to promote credibility to encourage use of the website. Participants were invited to read about the expertise of content developers. The website logo reflects the design of awareness ribbons often associated with cancer awareness. The university colors (white, purple, and green) would be used in the logo [14]. Logos of the university and the cancer charity that cofunded the research straddle the website logo.

The findings of the preparatory qualitative research, therefore, led to the development of design objectives which were consulted throughout the planning and development phases. This helped to ensure that the intervention was founded on a consistent rationale that would optimize its acceptability, feasibility, and in turn, effectiveness. With this user-oriented approach to design, the developers of the intervention were able to access information that complemented the application of psychological theory in the design of the program.

#### Application of Psychological Theory in Design Process

Psychological theory was also used to inform the optimal implementation of different design features and BCTs within different intervention contexts [58]. A list of intervention components resulting from the iterative process of applying principles and BCTs can be seen in Table 2.

Personalization was used throughout the website (eg, inserting a person’s name) as self-referent cues are believed to be important in encouraging effortful processing [58,61]. Strategies for providing choice and flexibility were included where possible to enhance users’ sense of autonomy [58]. Users were encouraged in every session to reflect on their own personal, intrinsic reasons for using the website and on how suggested changes could be incorporated into their lives [56]. The use of vignettes and quotes from the focus groups was incorporated to meet users’ need for relatedness in the hope that users would feel listened to and by recognizing the challenges faced by CrF [58]. Stories from similar others, including reflections on how to cope with fatigue, aimed to develop a sense of self-efficacy through vicarious experiences. A sense of relatedness was promoted using videos and by introducing the research team via a “meet the team” page. Competence was promoted by encouragement, gradual increases in task difficulty, and available support from the team if the users had any questions [58].

A significant portion of the second session was devoted to goal-setting and learning to avoid the pursuit of inappropriate goals [62]. A “goal step-ladder” was introduced to participants to encourage the selection of sufficiently challenging and achievable goals that were linked to a longer-term distal goal [34,63]. As users progress through the sessions, the content changed from specific issues associated with fatigue management to broader issues associated with life after cancer [58,64].

As the intervention content was primarily focused on the self-regulation model theory, participants were encouraged to evaluate and reflect upon how planned or actual behavior directly affects fatigue, with framing and reframing of beliefs occurring throughout the sessions [58,65]. The use of a fatigue diary to monitor fatigue and understand its patterns was incorporated to assist participants in recognizing their symptoms. The sessions on negative mood, stress management, and relaxation provided skills-training to enhance a sense of control over the symptoms [7,66]. Participants were encouraged to actively appraise their cognitive-behavioral responses to symptoms throughout the sessions, and also in the phone calls with the intervention (for further information, see the study protocol [14]).

**Table 2:** Principles of website design and associated behavior change techniques (BCTs) included to promote the use of the “Refresh” program.

Principles of website design	Behavior change techniques
Social Support	3.1. Social support (unspecified)
	3.2. Social support (practical)
	3.3. Social support (emotional)
	6.3. Information about others’ approval
	12.2. Restructuring the social environment
Autonomy	2.1. Monitoring of behavior by others without feedback
	10.7. Self-incentive
	10.9. Self-reward
Goal setting	1.1. Goal setting (behavior)
	1.2. Problem solving
	1.3. Goal setting (outcome)
	1.4. Action planning
	1.5. Review behavior goals
	1.7. Review outcome goals
Self-monitoring	2.3. Self-monitoring of behavior
	2.4. Self-monitoring of outcomes of behavior
	5.4. Monitoring of emotional consequences
	12.5. Adding objects to the environment
Self-efficacy	6.3. Information about others’ approval
	10.4. Social reward
	14.4. Reward approximation
	15.1. Verbal persuasion about capability
Personalization	7.1. Prompts or cues
Normalizing symptoms	5.1. Information about health consequences
	5.2. Salience of consequences
	5.3. Information about social and environmental consequences
	5.6. Information about emotional consequences
	6.2. Social comparison
	4.3. Re-attribution
Focus on abilities	15.3. Focus on past success
	16.3. Vicarious consequences
	8.6. Generalization of target behavior
	8.7. Graded tasks
Skills-focused	4.1. Instruction on how to perform the behavior
	4.2. Information about Antecedents
	6.1. Demonstration of the behavior
	8.1. Behavioral practice or rehearsal
	8.2. Behavior substitution
	8.3. Habit formation
	8.4. Habit reversal
Length of the sessions	7.1. Prompts or cues
Credibility	9.1. Credible source

## Results

The findings of the preparatory deductive and inductive research were collated to create a plan of what the Web-based intervention should contain [19]. The following paragraphs describe the process of developing the intervention based on the results of this preparatory work. Factors such as website structure, views of stakeholders and how to present the content were considered before a version of the website was tested for usability.

### Part 3: Developing Web-Based Materials

The development process was informed by academics, clinical psychologists, and health psychologists having experience working with individuals affected by CrF, cancer, or fatigue. Specialists in the development and evaluation of Web-based behavior change interventions were also consulted. These included individuals with expertise in the design and implementation of interventions built using LifeGuide open-source software [67]. In order to ensure that an acceptable and feasible intervention was developed, the views of stakeholders, such as health care staff were also considered [13,68]. These included cancer care workers and staff at a local cancer support center. These consultations helped us to anticipate factors external to the intervention that may act as a barrier or facilitator to its implementation, or its effectiveness [20]. These included issues relating to computer literacy, the burden of fatigue, and potential preferences for offline support in this user group. We sought to design the website so that it would be easy to use and understand, with these considered as key factors in initial feasibility testing [14]. We also decided to use a variety of recruitment strategies to target individuals who were most likely to engage with a Web-based intervention (ie, through social media as well as through traditional recruitment methods) [14].

A draft content manual and plan for the structure of the Web-based intervention were designed. Due to the nature of eHealth interventions, certain aspects of the content manual could not be translated as originally planned. For example, some paragraphs were replaced with diagrams as shorter text was required to make the website more visually appealing. A storyboard was made for each session to demonstrate how the information would be presented on each Web page. Time and staffing resources were limited and so certain aspects of the content were prioritized by the research team [13]. These were based on the theoretical underpinnings of the research, as depicted in the logic model. Other aspects were altered or delivered in a different way than originally planned and some features that were not deemed essential were removed (eg, superfluous messages that did not include a BCT) [13,69]. An iterative review process then took place with the design team examining the different sessions. The original offline manual was useful as the website was extensively tunneled and tailored throughout this process, as with similar interventions (eg, Michie et al) [19].

All pages were created in Life-Guide’s virtual research environment (VRE) [70]. This allowed the team to share Web-based feedback, comments, and suggested amendments on each of the pages. Employing testing methods that allow for the exploration of user experiences allows researchers to better understand the processes involved [13,19,56].

#### Intervention Structure

According to Danaher, McKay, and Seeley [71], the information architecture (IA)—the structure of website information—is a key factor that is often overlooked in the design of behavior change websites. The “Refresh” program utilized a hybrid IA design. The layout allowed for easy navigation to each of the main sections of the site. This design was in line with user preferences as it allowed the individual to explore content weekly sessions outside the main intervention while still maintaining the focused forward movement of the tunnel program [71].

The user would begin by accessing an initial Web page that contains a welcome and access to a sign-up page (see Multimedia Appendix 1)). Logging in enabled access to a page that provided matrix-like access to 4 content areas (see Multimedia Appendix 2). Once logged in, each user was presented with a personalized home page that provided information about the last time the user logged in. The user had free access to 5 different pages from the home page (a matrix design; see Multimedia Appendix 3). This matrix design was also used on the optional pages that facilitated autonomy by allowing interested users to seek out supplementary information about the program if they wished to do so [71].

The 8 sessions of the intervention were similar to the weekly sessions conducted in traditional in-person CBT [14]. Given the structured nature of traditional CBT, some tunneling was necessary. The pages that used a tunnel design require few navigational controls other than the “back” and “next” buttons. A linear model was better suited for multisession programs in which users were assigned tasks to do in between Web-based sessions. This model also allowed for an incremental increase in the amount of information and BCTs that a user was exposed to, increasing the likelihood that the user learned and potentially used the strategies. Further information about the procedure of the intervention is published elsewhere [14].

Ancillary pages in the hybrid design could enable the user to customize their experience, seeking out extra information if they chose and not being constrained by the tunnel design. Ancillary pages provided links to Web page resources outside of the program; however, these were programmed to open in a new tab to ensure that users did not need to leave the website to gain extra information. Participants’ answers were saved to reload at the end of each page so that participants could pick up where they left off if they have to log out or take a break during a session. This design was used to facilitate user autonomy.

Hybrid designs offer the user alternative (and potentially more engaging) ways of interacting with, or revisiting content [71]. It was decided that this structure would be attractive as well as usable based on the reported preferences of participants in the qualitative study.

#### Presentation of Content

The sessions were short in length and a brief amount of text was displayed on each page. People do not tend to read long pages of text in Web-based interventions [72]. Participants often scan the page, picking out individual words, sentences, or images. To improve clarity, short concise sentences were presented in large, clear font styles. Text was chunked into short paragraphs to make the page feel less text-heavy. Lots of empty space (eg, between borders and text) and bullet pointed message were used to break up text. Bold font was used to highlight the main points on the page, with main points at the top of the page. Attempts were made to fit what needs to be conveyed on a page so that end users would not need to scroll down if possible. To break up text and reinforce meaning, as well as to reduce monotony, a variety of media were used to deliver the content. These included illustrations, text, animated videos with music and voiceovers, and the use of vignettes based on testimonials from qualitative research participants [73].

### Part 4: Usability Testing

Usability testing was employed to further develop and improve the website by assessing preliminary functionality, acceptability, usability, and engagement [19,72]. The data was analyzed to examine beliefs of the users and information about specific content, format, and navigation-related feedback. This feedback was used to modify the relevant components of the intervention [19,72].

Users were asked to “think aloud” to enable the team to identify problems people might experience when working through the intervention (eg, navigational difficulties or potential adverse reactions). Participants (a testicular cancer survivor and a nurse) interacted with functional draft Web pages and asked to comment on their reactions to every aspect of the intervention, focusing on the helpfulness of information provided, comprehension, and ease of use [56]. They were asked to describe what they liked or disliked, or if there were any aspects of the intervention that they would change. The findings are summarized in Textbox 1.

Other participants used the intervention alone as an end user and completed a survey about their experiences after completing some or all of the intervention. These participants included a cancer care assistant, a spouse of a cancer survivor with fatigue, and 2 PhD students studying health psychology. This was to gather information about how people use the program in the absence of a researcher. Again, participants were asked to note any aspects that they found particularly beneficial or not useful, easy to use or problematic, and aspects which they particularly enjoyed or disliked [13].

The team encouraged users to provide critical feedback to guide improvements to the program [56,74]. Major changes to the intervention were not required at this stage. Some minor modiﬁcations were incorporated, and pages were redrafted (see Textbox 1). At this stage, the primary aim was to establish usability. Assessment of user satisfaction and acceptability will be conducted with a sample of posttreatment cancer survivors in the pilot trial [14].

**Changes to website design identified by user-testing.**

Changes

Change bright purple border around buttons. Use darker shade.

- Use of bold font to emphasise key points and improve design.
- Fix formatting issues relating to content layout.
- Some videos not working, voiceover volume low.
- Include an instruction video to introduce the site.
- Change unhelpful jargon and terminology.
- Some typos identified.
- Remind people to scroll down on pages where it is necessary to do so.
- Email reminder should contain a link to the website for easy access.
- Ensure that email reminders are sent on time.
- Increase font size in some parts of the website.

## Discussion

### Principal Findings

This paper describes the development of “Refresh,” a Web-based, CBT-based intervention for CrF after the completion of cancer treatment. The intervention was developed through the systematic application of theory, evidence, and user-testing [19]. Despite being a complex and multifaceted intervention, transparency was sought by detailing the components of the intervention, the proposed mechanisms of change. Efforts were made to reduce the “black box” criticism of interventions [15,19] by offering a clear description of the intended intervention, and how it is expected to work, before its evaluation [20].

The cocreation of the intervention with the experts and service users allowed the design team to ensure that an acceptable intervention was developed. Involving users from the target group at the design stage can signiﬁcantly contribute to the development of interventions by highlighting aspects of the design that would have otherwise been missed [75,76]. However, due to time and financial constraints, it was not always possible to involve users as much as we would have hoped. Final testing of the website was carried out by colleagues in some cases, rather than individuals with fatigue. Testing the website with the target audience could improve implementation by further considering the burden of using the website and the levels of computer literacy required. We are keen to explore this further in our feasibility and pilot trials of the website [14].

Acknowledging the limitations of our design process, we therefore suggest that our method could potentially serve as a template, with the hope that researchers would continue to develop and refine this process.

### Conclusions

This evidence-based Web-based program is the ﬁrst intervention of its kind based on the self-regulation model theory, with the primary aim of targeting the representations of fatigue and enhancing self-management of CrF, speciﬁcally [33]. In line with the Medical Research Council (MRC) guidelines, the use of theory in developing the content was predicted to facilitate understanding of the causal assumptions underpinning the intervention [15]. The use of theory also reflects recent research which recognizes self-management as essential components for recovery of health and well-being in cancer survivorship [7,77].

The development of the intervention was informed by the MRC guidelines on developing complex interventions [15]. There is a need for the publication of more detailed descriptions of foundations that underpin complex interventions, promoting methodological rigor, and transparency in the design process [15,78]. This research sought to integrate psychological theory, existing evidence of effective interventions, empirically derived principles of Web design, and the views of potential users into the systematic planning and design of the intervention of an easy to use website for cancer survivors [1,5,7,19,75].

## Appendix

Multimedia Appendix 1 Welcome screen of "Refresh" intervention.

Multimedia Appendix 2 Login Sucess Screen of the "Refresh" intervention.

Multimedia Appendix 3 Homepage of the "Refresh" intervention.

## Abbreviations

BCT: behavioral chenge technique
CBT: cognitive behavioral therapy
CrF: cancer-related fatigue
MS: multiple sclerosis
VRE: virtual research environment
