# Supplementary figures and images for: Cancer-Related Fatigue in Post-Treatment Cancer Survivors: Theory-Based Development of a Web-Based Intervention
Source: JMIR Cancer. 2017 Jul 4;3(2):e8. doi: 10.2196/cancer.6987 (PMC5516102; doi:10.2196/cancer.6987)

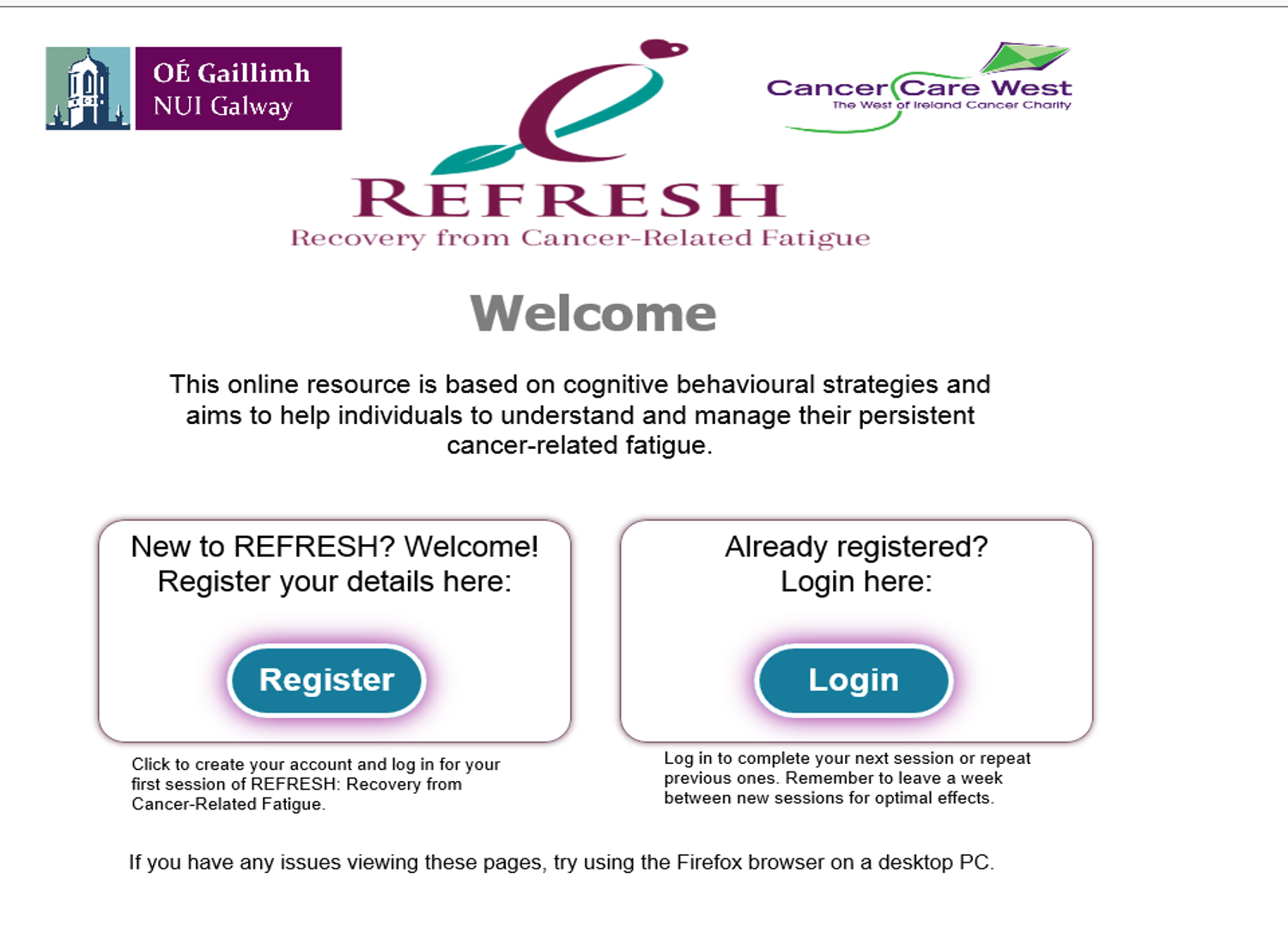

Supplement: Multimedia Appendix 1 [file cancer_v3i2e8_app1.png]

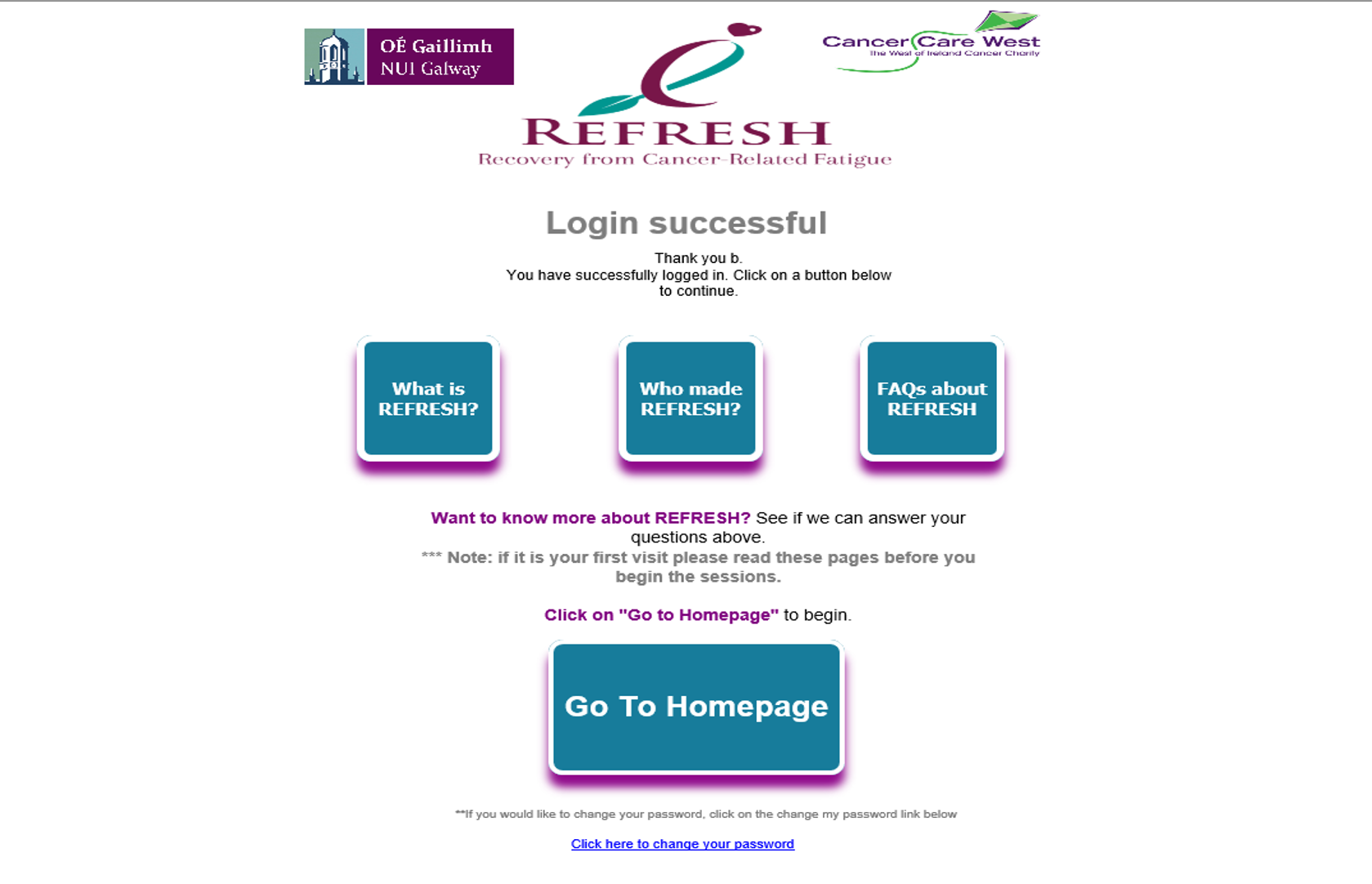

Supplement: Multimedia Appendix 2 [file cancer_v3i2e8_app2.png]

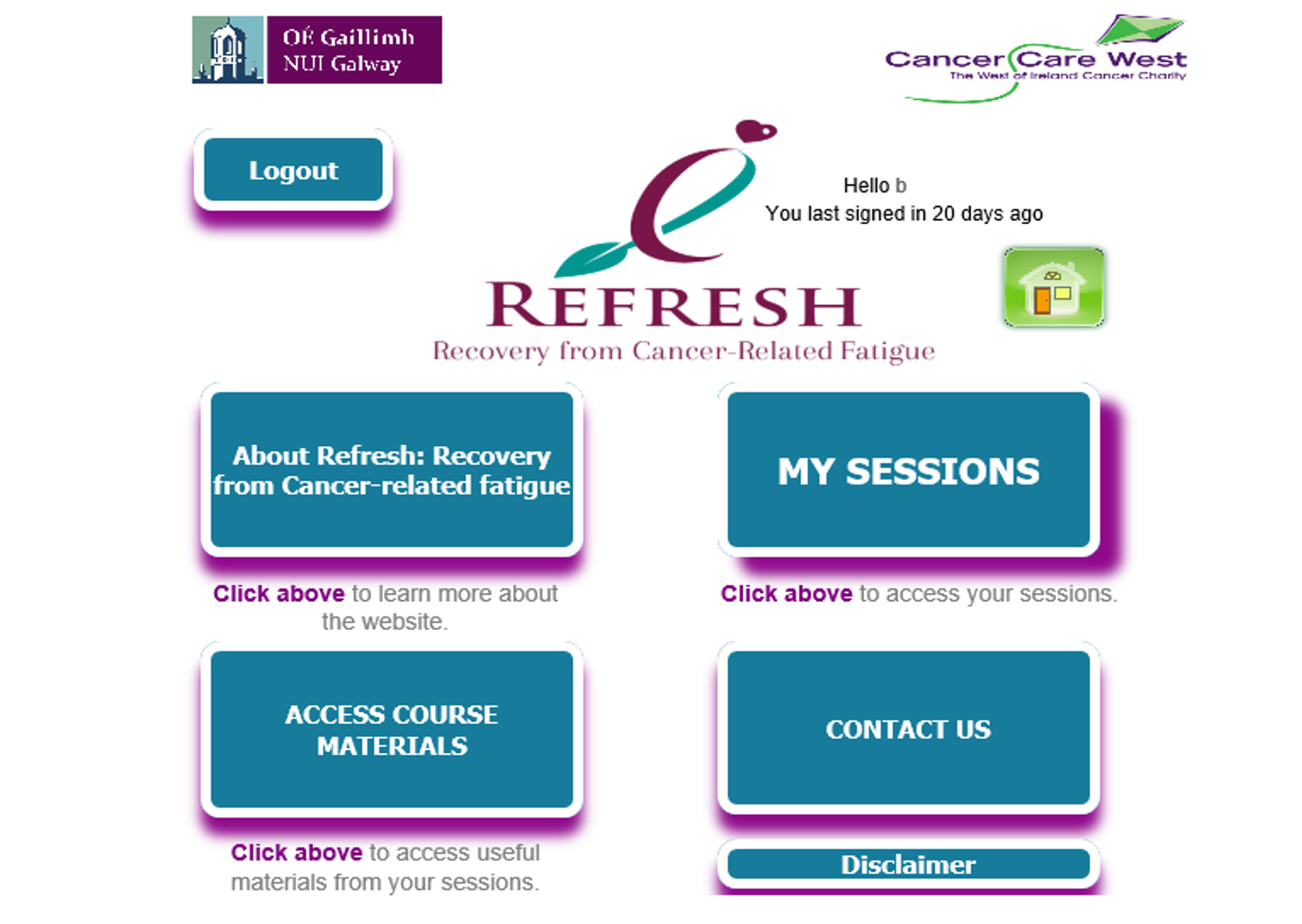

Supplement: Multimedia Appendix 3 [file cancer_v3i2e8_app3.png]
